# Transcript Specificity in Yeast Pre-mRNA Splicing Revealed by Mutations in Core Spliceosomal Components

**DOI:** 10.1371/journal.pbio.0050090

**Published:** 2007-03-27

**Authors:** Jeffrey A Pleiss, Gregg B Whitworth, Megan Bergkessel, Christine Guthrie

**Affiliations:** Department of Biochemistry and Biophysics, University of California San Francisco, San Francisco, California, United States of America; University of California Los Angeles, United States of America

## Abstract

Appropriate expression of most eukaryotic genes requires the removal of introns from their pre–messenger RNAs (pre-mRNAs), a process catalyzed by the spliceosome. In higher eukaryotes a large family of auxiliary factors known as SR proteins can improve the splicing efficiency of transcripts containing suboptimal splice sites by interacting with distinct sequences present in those pre-mRNAs. The yeast Saccharomyces cerevisiae lacks functional equivalents of most of these factors; thus, it has been unclear whether the spliceosome could effectively distinguish among transcripts. To address this question, we have used a microarray-based approach to examine the effects of mutations in 18 highly conserved core components of the spliceosomal machinery. The kinetic profiles reveal clear differences in the splicing defects of particular pre-mRNA substrates. Most notably, the behaviors of ribosomal protein gene transcripts are generally distinct from other intron-containing transcripts in response to several spliceosomal mutations. However, dramatically different behaviors can be seen for some pairs of transcripts encoding ribosomal protein gene paralogs, suggesting that the spliceosome can readily distinguish between otherwise highly similar pre-mRNAs. The ability of the spliceosome to distinguish among its different substrates may therefore offer an important opportunity for yeast to regulate gene expression in a transcript-dependent fashion. Given the high level of conservation of core spliceosomal components across eukaryotes, we expect that these results will significantly impact our understanding of how regulated splicing is controlled in higher eukaryotes as well.

## Author Summary

The spliceosome is a large RNA-protein machine responsible for removing the noncoding (intron) sequences that interrupt eukaryotic genes. Nearly everything known about the behavior of this machine has been based on the analysis of only a handful of genes, despite the fact that individual introns vary greatly in both size and sequence. Here we have utilized a microarray-based platform that allows us to simultaneously examine the behavior of all intron-containing genes in the budding yeast *S. cerevisiae.* By systematically examining the effects of individual mutants in the spliceosome on the splicing of all substrates, we have uncovered a surprisingly complex relationship between the spliceosome and its full complement of substrates. Contrary to the idea that the spliceosome engages in “generic” interactions with all intron-containing substrates in the cell, our results show that the identity of the transcript can differentially affect splicing efficiency when the machine is subtly perturbed. We propose that the wild-type spliceosome can also distinguish among its many substrates as external conditions warrant to function as a specific regulator of gene expression.

## Abstract 3

Many eukaryotic gene transcripts are spliced; here the authors show that components of the splicing complex can distinguish between different introns in highly homologous transcripts.

## Introduction

The coding regions of most eukaryotic genes are interrupted by introns, which must be removed for proper gene expression. The removal of introns requires single-nucleotide precision in order to faithfully convert genomic information into functional protein. The process of intron removal is performed by the spliceosome, a large ribonucleoprotein that catalyzes two sequential transesterification reactions [1,2]. The spliceosome itself is highly conserved across all eukaryotes, consisting of five small nuclear RNAs (snRNAs) and well over 100 proteins [3,4]. A combination of genetic and biochemical experiments have revealed conformational rearrangements in both RNA and protein components that are required for the spliceosome to accurately process pre–messenger RNA (pre-mRNA) transcripts [5]. About 50 of these proteins can be considered core components of the spliceosome in that their activity is required for cell viability in budding yeast, whereas the remainder of the factors can be deleted with little or no effect on cell growth under standard laboratory conditions.

To date, the vast majority of what is known about the mechanism of pre-mRNA splicing has been deduced from experiments using, at most, a handful of transcripts. Yet it remains unknown how well these transcripts represent the behavior of the entire complement of spliceosomal substrates. In higher eukaryotes, where genes are often interrupted by multiple introns, it is known that the spliceosome can utilize specific sequences present in individual transcripts to regulate both quantitative and qualitative aspects of gene expression [6,7]. Two large groups of proteins, the SR and hnRNP families, are known to modulate splicing activity, allowing the spliceosome to generate multiple distinct proteins from a single genomic locus, significantly increasing proteomic diversity. In particular, the sequence-specific RNA-binding members of the SR family improve the processing efficiency of introns containing suboptimal signals at their 5′ or 3′ splice sites. Binding of the SR proteins to enhancer sequences, which may be located in exons or introns, facilitates recruitment of the core machinery to the suboptimal splice sites.

**Figure 1:**
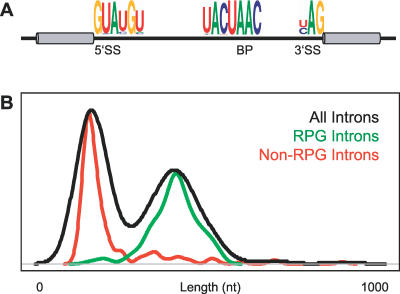
Properties of Yeast Introns (A) Consensus sequences found in yeast introns at 5′ splice sites (5′SS), 3′ splice sites (3′SS), and branch points (BP) [37]. (B) Distribution of yeast intron lengths shown for all introns (black), introns only in ribosomal protein genes (green), or introns only in nonribosomal protein genes (red).

By comparison to higher eukaryotes, splicing in the yeast Saccharomyces cerevisiae appears much simpler in a number of ways. Whereas more than 95% of human genes are interrupted by an intron [8], only about 250 yeast genes, or less than 5% of all genes, contain an intron [9]. Moreover, the vast majority of the intron-containing genes in yeast contain only a single intron, differing significantly from humans, where the average gene is interrupted by eight introns [8]. As seen in Figure 1A, yeast introns tend to conform to very strong consensus sequences at the branch point and at both the 5′ and 3′ splice sites. Perhaps accordingly, only three SR-like proteins have been identified in yeast, none of which is known to regulate splicing activity. On the other hand, while relatively few yeast genes are interrupted by introns, those that are tend to be highly expressed and tightly regulated genes. It has been previously shown, for example, that intron-containing genes account for nearly one-third of total cellular transcription [10]. Notably, several functional categories are overrepresented among intron-containing genes. The most dramatic of these corresponds to transcripts that encode ribosomal protein genes (RPGs): 102 of the 139 RPGs in yeast contain introns. Interestingly, all yeast introns tend to be small relative to those found in humans; the largest yeast intron contains only 1,002 nucleotides, whereas many human introns stretch to well over 10,000 nucleotides. Nevertheless, there is a bimodal distribution of intron lengths that has a strong correlation with gene function; introns found in genes encoding ribosomal proteins tend to be longer, while those in nonribosomal proteins are generally shorter (Figure 1B).

Given the relative simplicity and similarity of intron-containing transcripts in yeast and the limited role of the SR proteins, it has been a reasonable expectation that the yeast spliceosome would have a restricted capacity to distinguish among its different intron-containing substrates. In this case, the behavior of a few individual transcripts could be extrapolated to accurately describe the behavior of most spliceosomal substrates. The advent of splicing-specific microarrays, which facilitate a simultaneous examination of all intron-containing transcripts [11], allows us to directly ask whether the spliceosome interacts similarly with all pre-mRNAs, or whether transcript-specific differences can be identified. Here we have used this approach to examine the splicing responses of all ∼250 intron-containing transcripts resulting from inactivation of 18 different core components of the spliceosome and five factors involved in up- or downstream steps in pre-mRNA processing. Far from being homogeneous in their effects on the different intron-containing transcripts, these mutants reveal a remarkable ability of the core components to differentiate among transcripts. Interestingly, most ribosomal proteins are encoded at two distinct genomic loci in yeast. Whereas the coding sequences are nearly identical at these two locations, the sequences of the introns that interrupt them are generally quite dissimilar. For some of these paralogs, dramatic differences can be seen in response to the different spliceosomal mutations. The capacity of the spliceosome to differentiate among substrates suggests that splicing offers an important opportunity to regulate gene expression in a transcript-specific manner.

## Results

### Monitoring Genome-Wide Changes in Splicing

**Figure 2:**
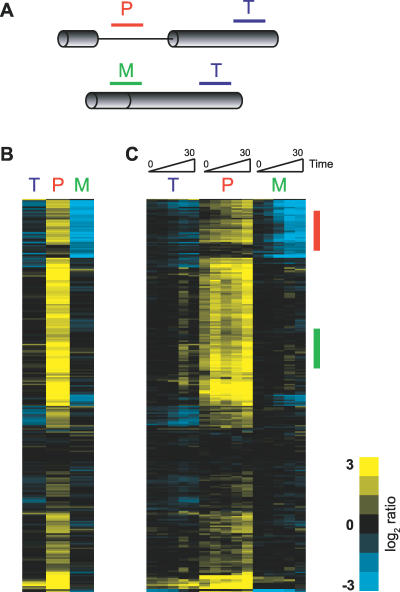
Monitoring Genome-Wide Changes in Pre-mRNA Splicing (A) Oligos target either the pre-mRNA (P), the mature mRNA (M), or the total level of mRNA (T) for each intron-containing gene. (B) False color representation of the splicing response of 249 different intron-containing transcripts at a single timepoint after inactivation of Prp2 activity. Each horizontal line describes the behavior of a single transcript. (C) Time course from 0 to 30 min showing the kinetic response to inactivation of Prp2. The gene order is identical to that shown in (B).

To examine genome-wide changes in splicing in yeast, we have designed microarrays similar to those previously described [11] that allow us to distinguish between the spliced and unspliced isoforms of transcripts derived from intron-containing genes. As seen in Figure 2A, three different oligos are used to target each intron-containing gene (see also Materials and Methods). One oligo targets a portion of the last exon and is used to detect changes in total transcript level (probe T). A second oligo targets a region of the intron, allowing us to detect changes in pre-mRNA levels (probe P), while the third oligo targets the junction of the two exons, allowing for a determination of changes in levels of mature mRNA (probe M).

Because there are multiple, independent probes describing the behavior of each intron-containing gene, analysis of a splicing-specific microarray is inherently more complicated than that of a standard expression array. In order to assess the splicing behavior of any given transcript it is important to simultaneously examine the changes in signal on all three feature types. In analyzing data from splicing-specific microarrays, others have generated splicing indices for each gene by compressing the data derived from the different feature types. For example, splicing changes can be represented as a precursor/mature (PM) index by dividing the ratio of the movement of the pre-mRNA feature by the ratio of the mature mRNA feature [11,12]. Although this manipulation simplifies the data, allowing them to be more easily examined in the context of traditional microarray analyses, for reasons described below we find that a more detailed understanding of individual transcript behavior is achieved by considering the independent movement of all three feature types simultaneously.

Figures 2B and 2C show an example of such an analysis derived from a comparison of RNA from a wild-type strain and a strain harboring the temperature-sensitive *prp2-1* mutation after both were shifted to the nonpermissive temperature of 37 °C. The spliceosomal Prp2 protein is a member of the DEAD/H box family of RNA-dependent ATPases and plays an essential role in catalyzing an as yet unidentified structural rearrangement required for activation of the spliceosome prior to the first chemical step [13]. As such, the primary result of a defect in Prp2 activity is expected to be a broad decrease in pre-mRNA splicing. The splicing profile resulting from this experiment, where each horizontal line describes the behavior of a single gene, is displayed for both a single timepoint (Figure 2B) and across the time course of inactivation (Figure 2C). A simple prediction for the behavior of a given transcript in an experiment where the activity of a core spliceosomal component is impaired is that the precursor species would accumulate with a concomitant loss of the mature species. Indeed, for many of the transcripts, including those shown with a red bar in Figure 2C, this is precisely the pattern that is observed: precursor species are seen to accumulate to nearly ten times the normal level, and up to 90% of the mature mRNA is lost. For most of these genes, the behavior of the total mRNA probe closely follows that of the mature species, suggesting that the majority of the transcripts for these genes are present in their spliced forms. However, different profiles are apparent for many other genes. For example, many show robust accumulation of precursor species with very little decrease in mature mRNA, including those shown with a green bar in Figure 2C.

The different behaviors displayed by these two groups of transcripts presumably reveal important differences in the complex interplay of cellular machineries that define the steady-state levels of both the precursor and mature forms of each of these transcripts. For example, the rate of precursor accumulation observed for a given transcript depends upon, among other things, the transcription rate of that gene, the degradation rate of the transcript, the rate at which the transcript is normally spliced, and the extent to which its splicing is inhibited. Likewise, the decrease in mature mRNA is a function of both the extent of splicing inhibition and the decay rate of the mature form of that transcript. For those genes marked by the red bar in Figure 2C, the dramatic decrease in levels of mature mRNA may be explained by either a strong block to the production of mature species, a rapid rate of decay of the mature species, or some combination of both. By comparison, a less complete block to splicing or a slower rate of mRNA decay may explain the failure to detect significant decreases in mRNA for those genes marked with the green bar. Importantly, the process of creating splicing indices to describe the behavior of these transcripts in many cases eliminates the ability to differentiate between these scenarios. For example, genes which show both a modest increase in precursor levels and a modest decrease in mature mRNA levels generate nearly identical PM index values as do genes that show a strong precursor accumulation but little decrease in mature levels.

By simultaneously examining each of the feature types as described above, the global splicing profile resulting from the *prp2-1* mutation shows that precursors rapidly accumulate for about 80% of the intron-containing genes. While the detailed behaviors of the different transcripts are varied, this result appears to satisfy the simple prediction that defects in this factor would result in defective splicing of all actively transcribed genes. At least two different scenarios may explain the failure to detect precursor accumulation for the remaining 20% of the transcripts. It is almost certainly true that some of the intron-containing genes are transcriptionally quiescent during logarithmic growth conditions. Naturally, no precursor accumulation would be expected for such genes. Alternatively, these transcripts may be insensitive to the particular defect introduced by the *prp2-1* mutation.

### Transcript Specificity of Spliceosomal Mutations

In order to further distinguish between transcript behaviors, we examined the RNA from strains containing mutations in two additional core spliceosomal components: *PRP5* and *PRP8.* Like *PRP2, PRP5* encodes an essential member of the DEAD/H box family of RNA-dependent ATPases. By comparison with Prp2, the activity of Prp5 is required to promote a molecular rearrangement earlier in the splicing pathway, catalyzing the stable addition of the U2 small nuclear ribonucleoprotein to the branch point sequence [14]. As with mutations in Prp2, loss-of-function mutations in Prp5 result in a block to splicing prior to completion of the first chemical step. Notably, Prp5 and Prp2 are only transiently associated with the spliceosome, whereas Prp8 is a stable component of the U5 small nuclear ribonucleoprotein and is thought to form the physical core of the spliceosome [15,16]. As with Prp5 and Prp2, most mutations in Prp8 block spliceosomal activity prior to the first chemical step; however, mutants have been isolated that can perform the first but not the second transesterification reaction [17,18].

**Figure 3:**
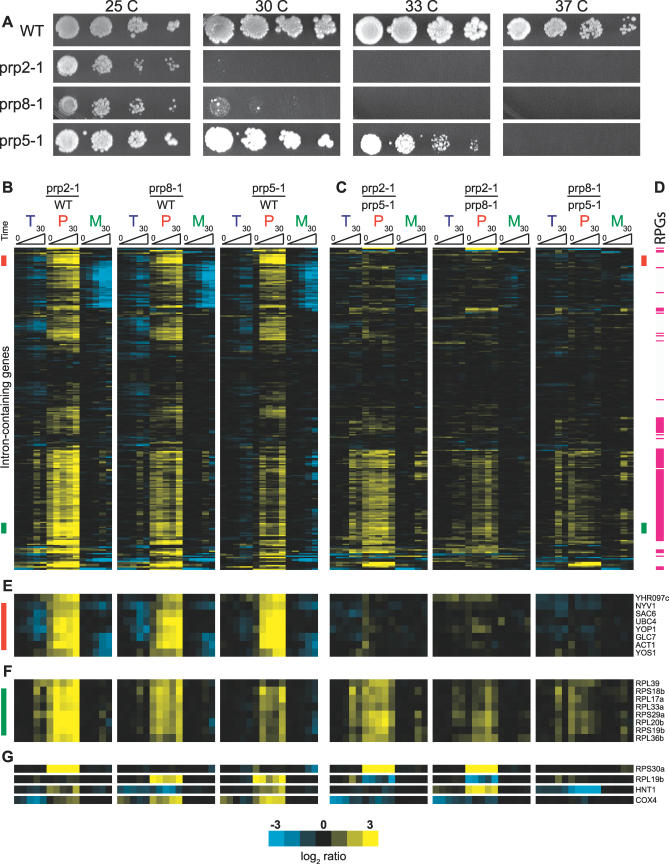
Comparing the Cellular and Molecular Phenotypes of Three Different Spliceosomal Mutants (A) Serial dilutions of a wild-type strain and strains containing the *prp2-1, prp8-1,* and *prp5-1* mutations are grown at the temperatures indicated. (B) Time-resolved splicing profiles for each of the three mutant strains compared to a wild-type strain. (C) Time-resolved profiles resulting from microarrays directly comparing the indicated mutants. The order of genes is identical to that in (B). (D) Transcripts that encode ribosomal protein genes are indicated with a red line. The order of genes is identical to that in (B) and (C). (E) The identification and behavior of a subset of genes indicated with a green bar in (B) and (C). For these transcripts the comparison experiments reveal a different level of precursor accumulation for each of the mutants. (F) The identification and behavior of a subset of genes indicated with a red bar in (B) and (C). For these transcripts the comparison experiments show an identical splicing defect for all three mutants. (G) The identification and behaviors of a subset of genes whose splicing is affected by only one or two of the three mutants studied.

As seen in Figure 3A, strains containing the loss-of-function mutations *prp2-1, prp8-1,* or *prp5-1* all exhibit conditional growth phenotypes, showing nearly wild-type growth at 25 °C but an inability to support growth at 37 °C. A comparison of growth at intermediate temperatures allows these mutations to be ordered according to the strength of the defect they impart: *prp2-1* is the most severe defect as it is unable to support growth above 25 °C; *prp8-1* is slightly less severe as it can support weak growth at 30 °C; and *prp5-1* is the weakest as it shows nearly wild-type growth even at 33 °C. Figure 3B shows the time-resolved splicing profiles derived from shifting each of these strains from 25 °C to 37 °C and comparing them to a similarly treated wild-type strain. Importantly, the ordering of the genes is consistent for all three profiles, meaning that the behavior of a single intron-containing gene can be seen by following a single horizontal line across all three profiles.

As expected, inactivation of each of these factors results in inhibition of splicing of a large number of intron-containing transcripts. Interestingly, while the overlapping set of transcripts that are affected by inactivation of all three factors is quite large, a closer examination suggested important differences in the particular transcripts whose splicing was affected and prompted us to more carefully compare the profiles of these mutants. Microarray experiments were performed that directly compared the pairwise behaviors of these mutant strains (Figure 3C), revealing three distinct classes of transcript behavior. The first class is composed of transcripts for which splicing is equally affected by each of these mutations. Approximately 100 transcripts behave in this fashion, exemplified by those indicated with a red bar in Figure 3B and 3C and highlighted in Figure 3E. For these transcripts, the comparison of mutant versus wild-type behavior seen in Figure 3B shows a strong splicing defect for all three mutations. Further, the direct comparisons of the mutants in Figure 3C show that each of the mutants causes a nearly identical level of precursor accumulation and mature mRNA reduction. Interestingly, found within this class is the transcript encoding Act1, which is widely used as a reporter for both in vivo and in vitro splicing assays. Figure S2 shows the results of quantitative RT-PCR experiments validating a subset of these findings.

The second class is also composed of transcripts that exhibit a canonical splicing defect in response to inactivation of each of the single mutants, but for whom the magnitude of the defect is different for each of the mutants. About 100 transcripts behave in this fashion, exemplified by those indicated with a green bar in Figure 3B and 3C and highlighted in Figure 3F. As with the first class, the experiments shown in Figure 3B demonstrate that the splicing of each of these transcripts is affected by each of the mutations; however, the direct comparisons of the mutants in Figure 3C show a larger precursor accumulation in the *prp2-1* strain than in either the *prp8-1* or *prp5-1* strains, and a larger accumulation in the *prp8-1* strain than in the *prp5-1* strain. Interestingly, the molecular splicing defects observed for this class of transcripts correlate with the conditional growth defects: the *prp2-1* mutation produces the strongest phenotype, followed by the *prp8-1* mutation, and finally the *prp5-1* mutation. Notably, as indicated in Figure 3D, the vast majority of the transcripts in this class encode RPGs, and most RPGs belong to this class.

The final class is composed of a smaller number of transcripts, those that exhibit splicing inhibition in response to one or two of the mutant strains but not all three. Examples of several transcripts belonging to this class are shown in Figure 3G. Interestingly, the splicing of the Rps30a transcript is dramatically affected by the *prp2-1* mutation, but shows little defect in response to either the *prp8-1* or *prp5-1* mutations. Conversely, the splicing of the Rpl19b transcript is affected by both the *prp8-1* and *prp5-1* mutations, but is largely unaffected by the *prp2-1* mutation. Likewise, the Hnt1 transcript shows little defect in response to the *prp8-1* mutation, while splicing of the Cox4 transcript is strongly affected by the *prp5-1* mutation but is only mildly affected in the *prp2-1* strain, the inverse of the ordering seen for the second class of transcripts described above.

Given the predominance of RPG transcripts in the second class of transcripts, we sought to identify properties of these transcripts that might distinguish them from the others. As an initial attempt to identify such differences, we asked whether RPG transcripts showed a difference in splicing efficiency relative to other transcripts under standard laboratory growth conditions in wild-type cells. Quantitative RT-PCR was performed using primers specific to both intron and exon regions of 12 different intron-containing genes. Using genomic DNA to generate standard curves, relative copy numbers of both the precursor and total mRNA species were then calculated and compared. As seen in Table 1, the RPG transcripts tend to show exceptionally high levels of splicing efficiency, particularly relative to non-RPG transcripts. The relative copy numbers for the total mRNA features is in good agreement with previously published mRNA abundance values [19]. Interestingly, while the mature RPG transcripts are quite abundant, there is in fact less detectable precursor species for most RPGs than for non-RPGs. Nevertheless, as seen in Figure S3, the ability to detect large increases in precursor levels on our microarrays is independent of the initial level of precursor mRNA.

**Table 1:**
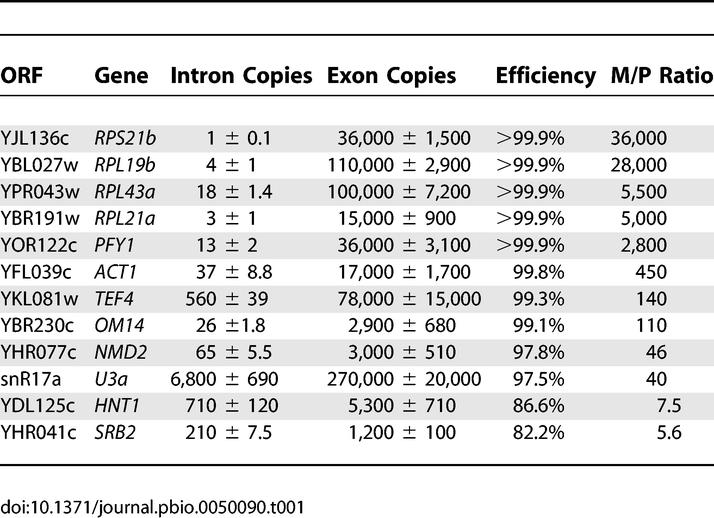
Relative Copy Numbers of Total and Pre-mRNA Determined by Quantitative RT-PCR

**Figure 4:**
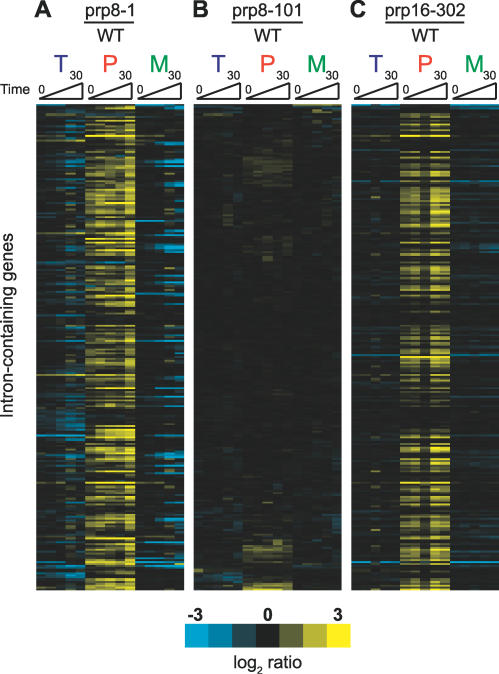
Two Different Alleles of Prp8 Produce Very Different Molecular Phenotypes Time-resolved splicing profiles derived from comparisons of *prp8-1* (A), *prp8-101* (B), or *prp16-302* (C), each compared to a wild-type strain. The order of the genes is the same for all three profiles.

The differences that we observed between mutations in different spliceosomal factors led us to ask whether distinct mutations in a single factor might also cause such transcript-specific responses. Shown in Figure 4A and 4B are the time-resolved splicing profiles obtained from shifting strains harboring either the *prp8-1* mutation or the temperature-sensitive *prp8-101* mutation, respectively, to the nonpermissive temperature and compared to similarly treated wild-type strains. Strikingly, the splicing of most intron-containing transcripts is almost completely unaffected by the *prp8-101* mutation. Instead, this mutation appears to affect the splicing of only a small number of transcripts. As it had previously been shown that the *prp8-101* mutation results in a defect in catalyzing the second transesterification reaction [18], we examined a defect in another factor necessary for the second step, the helicase Prp16 [20]. Figure 4C shows the time-resolved splicing profile derived from shifting a strain harboring the cold-sensitive *prp16-302* mutation to the nonpermissive temperature of 16 °C. The broad splicing defect seen for this mutant suggests that the differences between the *prp8-1* and *prp8-101* splicing profiles are not simply the result of comparing mutants defective for the first or second chemical steps, but rather truly reflect allele-specific differences in substrate specificity.

### A Systematic Examination of Splicing Factors

**Figure 5:**
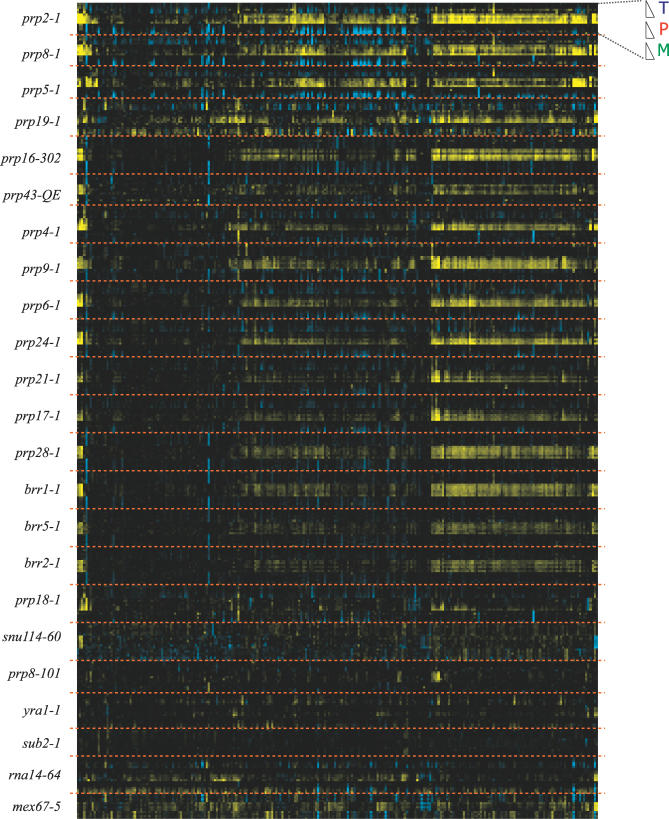
A Global View of Defects in Pre-mRNA Splicing Time-resolved splicing profiles derived from a panel of factors involved in pre-mRNA processing. The behavior of each individual transcript can be seen by following a vertical line. The particular mutations examined are shown on the left. For each mutant, the order is indicated at the top right corner of the figure.

The transcript-specific phenotypes that we observed with this small subset of conditional mutants compelled us to examine the effects of a much wider variety of spliceosomal defects. To facilitate these experiments, a set of high-throughput methods was developed that allowed us to automate many of the steps in a microarray experiment (see Materials and Methods). Using these methods, we examined the effects of 18 different mutations in core spliceosomal components and five additional mutations in up- or downstream processes in mRNA processing. Figure S4 shows the different mutants that were examined using these methods, the step in pre-mRNA processing at which they are presumed to be defective, and their conditional growth phenotype. Two different views of the data resulting from these experiments are presented in Figure 5 and Figure S5. By clustering the data across all of the experiments, Figure 5 tends to highlight those pre-mRNAs for which splicing is negatively affected by most of the mutants. By comparison, the clusters derived from each individual mutant shown in Figure S5 allow a more detailed analysis of the behavior of the pre-mRNAs in response to each individual mutation. As expected, many of the spliceosomal mutations result in splicing defects over a broad range of transcripts. Interestingly, the splicing profile resulting from inactivation of the *brr5-1* mutant is quite similar to that seen for many other canonical splicing mutants. While this mutant was originally isolated as a splicing mutant [21], it has been subsequently shown that Brr5 functions during 3′ end processing and is the yeast homolog of mammalian CPSF-73 [22,23]. By comparison, the *rna14-64* mutant, which is also defective in 3′ end formation [38], produces a noticeably different phenotype.

**Figure 6:**
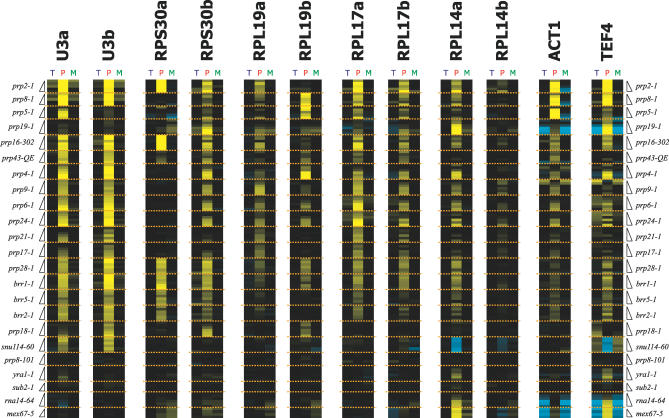
A Transcript-Specific View of Defects in Pre-mRNA Splicing Time-resolved splicing profiles for individual transcripts derived from the same panel of factors shown in Figure 5.

Importantly, a careful examination of the response of individual transcripts to this panel of mutations makes clear the fact that all transcripts are not equally affected by mutations in core components. To exemplify this point, Figure 6 shows the behavior of a select group of transcripts in response to all of the mutations examined. For some transcripts, such as the U3 small nucleolar RNA, splicing is rapidly blocked by nearly all spliceosomal mutations, while remaining largely unaffected by mutations in other mRNA processing factors. Interestingly, while the *prp19-1* mutation does affect the splicing of many pre-mRNAs, it has little effect on the splicing of this transcript. A comparison of the two U3 paralogs, *SNR17A* and *SNR17B,* whose mature products are nearly identical but whose introns share little sequence homology, also shows some differences in their responses to some of the splicing mutants. For example, the U3a transcript shows a stronger defect in response to the *prp5-1* mutation than the U3b transcript.

For some transcripts, the subset of mutations that affect their splicing is much different. A dramatic example of this is provided by the *RPS30* paralogs. Whereas splicing of the Rps30b transcript is affected by most of the splicing mutants examined, the Rps30a transcript is not. The splicing of the Rps30a transcript shows a strong defect in response to the *prp2-1* and *prp16-302* mutations, a somewhat weaker response to the *prp28-1, brr2-1, brr1-1,* and *brr5-1* mutations, and very little defect in most of the other mutants. The *RPL19* paralogs also display divergent behaviors: whereas the Rpl19a transcript shows a similar defect in response to each of the mutants, the Rpl19b transcript shows strong defects in response to the *prp8-1, prp5-1,* and *prp4-1* mutants, but less severe defects in response to the other mutants. Differences can also be seen between non-RPG transcripts by comparing the behavior of the Act1 and Tef4 transcripts, for example.

## Discussion

Here we describe the results of genome-wide experiments designed to examine the in vivo responses of all intron-containing transcripts in S. cerevisiae to mutations in core spliceosomal factors. For largely technical reasons, most experiments designed to study the efficiency of pre-mRNA splicing to date have focused on a relatively small number of transcripts, leaving open the question of how much variety there is among different spliceosomal substrates. The experiments presented here overcome this limitation by utilizing splicing-specific microarrays that allow for the simultaneous examination of all spliceosomal substrates. Splicing-specific microarrays are particularly amenable to studies in budding yeast, where there are a limited number of intron-containing genes and there is very little alternative splicing. By using this approach to examine the time-resolved effects of inactivating 23 different factors involved in pre-mRNA processing, the work presented here reveals a complex relationship between the activity of the core spliceosome and the full complement of transcripts with which it must interact.

### RPG Transcripts versus Non-RPG Transcripts

A large collection of data such as this offers a unique opportunity to both compare and contrast the behaviors of all 250 intron-containing transcripts according to their individual responses to the different spliceosomal mutations. From this perspective, the detailed comparison of mutations in *PRP2, PRP8,* and *PRP5* shown in Figure 3 provides important information about the different spliceosomal substrates, and suggests that all transcripts are not equally affected by particular spliceosomal mutations. Each of these three spliceosomal proteins plays an important role in catalyzing events necessary for the first chemical step in splicing. As such, mutations in these factors that reduce their activity at this step can be expected to negatively affect the splicing efficiency of all actively transcribed intron-containing genes. Indeed, while the majority of pre-mRNAs show such a defect in response to the *prp2-1, prp8-1,* and *prp5-1* mutations, the strengths of the splicing defects imparted by these mutations are variable. Whereas the splicing of many transcripts encoding factors other than ribosomal proteins is equally inhibited by mutations in any of these three factors, the strength of the defect observed in the splicing of most ribosomal protein gene transcripts is dependent upon the particular defect in the spliceosome. For these mutations, the strength of the molecular splicing defect correlates with the temperature sensitivity that the mutation imparts upon the strain: *prp2-1* causes the strongest defect and *prp5-1* causes the weakest. While the fraction of new transcript from any single gene whose splicing is inhibited cannot be determined from these experiments alone, these results nevertheless demonstrate that a greater fraction of most RPG transcripts is blocked by the *prp2-1* mutation than by either the *prp8-1* or *prp5-1* mutations. This difference is unlikely to simply reflect differences in the severity or onset of the protein mutations because nearly identical fractions of newly transcribed Act1, for example, are blocked by all three of the spliceosomal mutations. Rather, it suggests that the RPG-encoding transcripts are fundamentally different from the other transcripts in their susceptibility to these mutations.

We have not yet been able to identify any single feature of intron-containing RPGs that explains this altered susceptibility. While it is true that RPG introns tend to be longer than non-RPG introns, and that RPGs tend to be among the more highly transcribed genes in the genome, neither of these features alone seems sufficient to explain the different responses. The Act1 transcript, for example, is interrupted by an intron that is similar in length (308 nucleotides) to an average RPG intron (mean length of 402 nucleotides), and is transcribed at a similar rate as many RPGs [24], yet its behavior is distinct from that of most of the RPGs.

In an effort to identify properties that might differentiate RPG transcripts from other intron-containing transcripts, we used quantitative RT-PCR to determine the amount of precursor and mature mRNA species present in wild-type cells for a variety of different intron-containing genes. While all 12 of the transcripts that we examined were efficiently spliced (>80%), the RPG transcripts showed remarkably high partitioning toward mature species. It is noteworthy that, in spite of the high levels of total transcript present for most RPGs, the amount of precursor species present is remarkably low relative to the other intron-containing transcripts. For example, while there is more than ten times as much total Rps21b transcript in a cell as there is total Nmd2, 50 times less Rps21b pre-mRNA can be detected as compared to Nmd2 pre-mRNA.

It is tempting to speculate that the different splicing efficiencies and the different susceptibilities to spliceosomal mutations displayed by the RPGs may be mechanistically related. For example, while much recent progress has been made in identifying protein components of the spliceosome [4], it remains unknown whether the entire set of components is required for splicing of all transcripts, or whether different transcripts will utilize the activities of different subsets of factors. Given that the promoter elements driving transcription of RPGs are often different from non-RPGs, it is easy to imagine that the complement of proteins present during their splicing could also be different. The low levels of precursor species detected under steady-state conditions for the RPG transcripts suggest that the time between transcription and splicing is shorter for these transcripts than for non-RPG transcripts. In this light, it will be important to determine whether unique spliceosomal complexes are present during the splicing of RPG transcripts that allow for enhanced catalytic rates of either the first or second chemical steps of splicing. Alternatively, the rate of cotranscriptional loading of spliceosomal components onto RPG transcripts could be enhanced. In this case, genome-wide chromatin immunoprecipitation experiments might be expected to show higher levels of spliceosomal association with the RPGs than the non-RPGs. In either case, the presence or absence of unique, auxiliary spliceosomal components on the RPG transcripts might also change the susceptibility of these transcripts to defects in the core spliceosomal components.

### All RPGs Are Not Equal

Importantly, not all RPG transcripts behave similarly in these experiments. Whether considering the simple comparisons of the *prp2-1, prp8-1,* and *prp5-1* mutations, or the entire panel of factors as shown in Figures 5 and 6, several RPG transcripts show significantly different behaviors from the others. Compelling examples of these differences can be seen among certain pairs of RPG paralogs. While many paralogs display similar defects in response to the mutations, some pairs show dramatically different responses to these mutants (Figure 6). The *RPS30* paralogs offer the most dramatic example of these differences. Whereas splicing of the Rps30b transcript is adversely affected by almost all of the spliceosomal mutations that we examined, splicing of the Rps30a transcript is only affected by a small subset of these mutants. The particular features unique to each of these transcripts that underlie this differentiation remain unclear. Presumably the ability of the Rps30a transcript, for example, to be efficiently spliced in the *prp8-1* mutant does not indicate that Prp8 function is unnecessary for the splicing of this transcript. Rather, it suggests that the unique set of interactions that this transcript forms with the spliceosome are less sensitive to the defect imparted by this particular mutation in Prp8. Importantly, because the Rps30a and Rps30b transcripts behave so differently in response to so many mutations, they should prove to be extremely useful as tools for more traditional biochemical experiments designed to understand the function of individual spliceosomal factors.

### Deciphering Protein Function from Genome-Wide Experiments

Most of the mutants that we have examined here were isolated from conditional lethal screens. As such, this subset of mutations may be biased towards those with strong growth defects and hence broad transcript specificity. Perhaps because of this, we have found it difficult to associate a particular mechanistic role with any of these factors based simply on the subset of pre-mRNAs whose splicing is affected in these experiments. Rather, our findings seem to indicate that the signature of introns that are affected by a particular spliceosomal mutation is unique to that mutation. Nevertheless, it is tempting to imagine isolating other mutations in these core components that affect the splicing of a smaller subset of transcripts. Such mutations may be more difficult to isolate, as they may be aphenotypic for growth under standard laboratory conditions. Nevertheless, a genome-wide splicing analysis of such a mutant may provide important insights into the activity of the protein. Indeed, for the *prp8-101* mutation, which appears to represent such a mutant, knowing the identity of transcripts that are either strongly affected or unaffected provides important information for designing more traditional biochemical experiments in the future to examine the mechanism of its activity in the spliceosome. Likewise, the genome-wide splicing profiles derived from similar mutations in other factors might prove more revealing about the mechanistic role of those factors.

### Implications

Interestingly, the capacity of core spliceosomal components to elicit transcript-specific changes in splicing activity does not appear to be limited to yeast. Recent RNAi-mediated depletion experiments targeting core spliceosomal components in Drosophila melanogaster show differential effects on splicing activity at alternative splice sites [25]. Given the level of conservation of the core spliceosomal components across eukaryotes, it seems highly likely that they could also play important roles in augmenting the regulatory roles of the SR and hnRNP families of proteins in higher eukaryotes. The surprising finding that the macular degenerative disease retinitis pigmentosa is associated with mutations in core components of the spliceosome may in fact reflect such a role [26–28]. As seen here for the *prp8-101* mutation, the mutations associated with retinitis pigmentosa may cause defects in the splicing of a distinct group of transcripts, presumably uniquely associated with retinal function.

We have recently shown that the yeast spliceosome can rapidly and specifically alter the splicing efficiency of distinct subsets of transcripts in response to at least two unrelated environmental stresses (J.A.P., G.B.W., M.B. and C.G., unpublished data). Notably, in response to amino acid starvation, the splicing of virtually all RPG transcripts is specifically downregulated. Given that S. cerevisiae lacks the large SR and hnRNP protein families responsible for modulating transcript-specific splicing activity in higher eukaryotes, the observations presented in the current work suggest that the core spliceosomal components themselves could be targets of environmental regulation. As seen with stable genetic modifications here, post-translational modifications of these components might similarly facilitate transcript-specific regulation of pre-mRNA splicing. Such modifications would allow the cell to both rapidly and specifically regulate the production of translatable mRNA for particular transcripts in a reversible manner. Whether or not the complex relationships between transcripts and core spliceosomal components revealed by these mutant studies represent features of a system that has evolved to be the target of biological regulation is a provocative question that remains to be fully addressed.

The work presented here does however suggest that the relationship between the spliceosome and the full complement of transcripts with which it must interact is much more complex than previously believed. Important questions for the future concern both the mechanism by which the spliceosome distinguishes among these substrates and the biological rationale for this specificity. The simple hypothesis that all mutations in splicing factors that inhibit the growth of a yeast cell would also inhibit the splicing of all (expressed) intron-containing transcripts is inconsistent with our observations. The splicing efficiency of a given intron can not yet be ascribed to obvious *cis* features of the intron, nor does the biochemical activity presumed to be affected by a mutation appear to dictate which introns will be affected. Instead, in much the same way that transcription is now known to be regulated by the combinatorial control of its holoenzyme, it appears that the efficiency of pre-mRNA splicing may be dependent upon the complexes present in the spliceosome as well as the particular transcript upon which it assembles.

## Materials and Methods

### Sample collection and preparation.

Unless otherwise indicated, cultures were grown according to standard techniques in rich medium supplemented with 2% glucose at 25 °C. Both mutant strains and the corresponding wild-type strain were grown in parallel, 100 ml cultures until their optical densities were between A_600_ = 0.5 and A_600_ = 0.7. An initial 15 ml sample was collected at 25 °C prior to initiation of the time course. Cells were collected by filtration using Millipore HAWP0025 filters (http://www.millipore.com). The filters were immediately frozen in N_2_(l). The culture flasks were then transferred into water baths at either 16 °C or 37 °C, with additional 15 ml aliquots removed and collected at the appropriate times. Total cellular RNA was isolated as previously described [29] with a few exceptions. Tubes containing PhaseLock gel (Eppendorf, http://www.eppendorf.com) were used during phase separation steps. Also, RNA samples were precipitated using isopropanol.

For each microarray sample, cDNA was prepared from 25 μg of total RNA in a 50 μl reaction mixture containing 50 mM TrisHCl (pH 8.3), 75 mM KCl, 3 mM MgCl_2_, 10 mM DTT, 0.5 mM ATP, 0.5 mM CTP, 0.5 mM GTP, 0.3 mM TTP, 0.2 mM aa-dUTP, 12.5 μg dN_9_ primer, and 5 ng murine Moloney leukemia virus (M-MLV) RT. Primers were annealed to the RNA by heating to 65 °C for 5 min in the presence of buffer and salt alone. Reactions were allowed to incubate at 42 °C for at least 2 h. Remaining RNA was then hydrolyzed by incubation for 15 min at 65 °C in the presence of 0.1 M NaOH and 10 mM EDTA. This was neutralized with HCl, then purified using a Zymo25 DNA purification column according to the manufacturer's protocol (http://www.zymoresearch.com). Typical yields ranged from 10 to 15 μg of cDNA from 25 μg of starting material. The purified cDNA was then conjugated to the appropriate fluorescent dye in a 10 μl reaction containing 50 mM sodium bicarbonate (pH 9.0), 50% DMSO, and ∼20 μg of NHS-derivatized fluorophore. Reactions were incubated in the dark at 60 °C for 60 min, after which time the cDNA was repurified using a Zymo25 DNA purification column.

### High-throughput sample collection and preparation.

Cultures were grown in sets of four mutant strains along with their corresponding wild-type strains in rich medium in flasks at 25 °C until their optical densities were between A_600_ = 0.5 and A_600_ = 0.7. After the strains were synchronized, the cultures were transferred into the wells of a 96-well growth plate at 25 °C. A reverse time course was then initiated using a multichannel pipettor at the appropriate times to transfer the cultures into a second 96-well plate submerged in a 37 °C water bath (Figure S6). The entire time course was then collected by centrifugation of the plate at 5,000*g* for 5 min. The cell pellets were then frozen in N_2_(l). With the help of a Biomek FX liquid handling system (Beckman Coulter, http://www.beckmancoulter.com), total cellular RNA was then isolated using largely standard procedures. Typical yields from a single 1.8 ml well ranged from 15 μg to 25 μg of total RNA. For each well, cDNA synthesis was performed in a 50 μl reaction as described above. After cDNA synthesis, the remaining RNA was hydrolyzed by addition of 25 μl of 0.3 M NaOH/0.03 M EDTA and heating to 60 °C for 15 min. This solution was neutralized by addition of 25 μL of 0.3 M HCl. To purify the cDNA from this solution, 0.5 ml of cDNA binding buffer (5 M GdnHCl, 30% isopropanol, 60 mM potassium acetate, 90 mM acetic acid) was added to each well. This material was passed over a 96-well glass-fiber filtration plate (Nalgene Nunc, http://www.nalgenenunc.com). The filters were then washed once with 0.5 ml of Wash I (10 mM TrisHCl [pH 8.0], 80% EtOH), and once with 0.5 ml of Wash II (80% EtOH). Purified cDNA was then eluted in two steps of 100 μl water. The appropriate dyes were then coupled to the cDNA as described above. Fluorescently labeled cDNA was then repurified using 96-well glass-fiber filtration plates as described above.

### Quantitative PCR.

Total cellular RNA for quantitative RT-PCR experiments was prepared as described above. Prior to conversion to cDNA, the RNA was treated with DNase I (Fermentas, http://www.fermentas.com) according to the manufacturer's protocol. The cDNA was then produced from 2 μg of total RNA in a 20 μl reaction mixture containing 50 mM TrisHCl (pH 8.3), 75 mM KCl, 3 mM MgCl_2_, 10 mM DTT, 0.5 mM each dNTP, 25 nM each gene-specific primer, 2.5 μg dN_9_ primer, and 5 ng M-MLV RT. Quantitative PCR was then performed using an Opticon from MJ Research (http://www.mjresearch.com). Primer sets used in this study are shown in Table S1. Relative copy numbers shown in Table 1 are the result of triplicate measurements from a single biological sample. Standard deviations were only slightly higher when comparing biological replicates. A series of 10-fold dilutions of genomic DNA covering a total range of 10^6^ molecules was used to generate the standard curves. Genomic DNA was purified using a ZR Fungal DNA kit (Zymo Research) according to the manufacturer's protocol. The copy numbers presented in the Table have been normalized to the least abundant species detected in these experiments, the precursor isoform of the RPS21b transcript. Importantly, these copy numbers do not represent cellular copy numbers.

### Microarray design and construction.

In an initial set of experiments, we examined a variety of probe designs for their ability to specifically and efficiently identify the mature mRNA species derived from intron-containing genes. Whereas other splicing-specific microarrays have utilized fixed-length probes that are geometrically centered about the exon–exon junction, our best results were obtained when the probe was thermodynamically balanced between the two sides of the junction. Accordingly, oligonucleotides were designed for every exon–exon junction such that the hybridization energies were equivalent on either side of the junction (Figure S7). Because of the different GC contents in the transcripts, the junction oligos range in length from 28 nucleotides to 40 nucleotides.

Total mRNA feature probes (expression oligos) and premature mRNA feature probes (intron oligos) 32 nucleotides in length were designed using a previously described algorithm [30]. Intron and junction oligos were designed to target 256 intron features in 246 different transcripts. Expression oligos were designed to target the last exon of these 246 intron-containing transcripts, as well as 689 non–intron-containing transcripts and 99 noncoding RNAs (including snRNAs and tRNAs).

Probes were printed on poly-lysine-coated glass slides using standard techniques. Each probe was print on the array three times by each of two different printing pins (for a total of six replicate probe spots for each feature on each array; Figure S8). Prior to use, arrays were preprocessed by 60 s of incubation at 25 °C in 3× SSC, 0.2% SDS. Arrays were then blocked using standard procedures. Mutant and wild-type samples were then competitively hybridized against one another using the Agilent Hybridization Buffer (Agilent, http://www.agilent.com) at 60 °C for a period ranging from 12 to 16 h.

### Image analysis, data preprocessing, and higher order analysis.

Microarray images were acquired using an Axon Instruments GenePix 4000B scanner, reading at wavelengths of 635nm and 532nm (Axon Instruments, http://www.axon.com). Image analysis was performed using Axon Instruments GenePix Pro version 4.0 (number 2500–137, Rev E, 2001). Ratio values derived from the median pixel intensities for the 635 nm and 532 nm images of each spot were used to represent probe behaviors in data preprocessing analysis. A standardized qualitative assessment of array quality was performed using the Bioconductor arrayQuality package (Paquet AC, Yang YH, “arrayQuality: Assessing array quality on spotted arrays,” version 1.4.0) [31,32]. Arrays were manually removed from further analysis if they showed an above average spot intensity to ratio bias or a strong spatial ratio bias (Figure S9). Spot ratio data were log_2_ transformed and normalized within each array using global loess regression implemented in the Bioconductor marray package [33]. All experiments were performed as dye-flipped pairs using biological replicates. Both within-array and between-array data replication were analyzed for data quality (Figure S10) using the Bioconductor limma package [34]. Linear analyses of the data were performed using correlations of within-array and between-array spot replicates to assess evidence for differential expression of each RNA species across all of the experimental factors [35]. Results from these linear analyses pertaining to conclusions highlighted in the text are shown in Tables S2 and S3.

For downstream hierarchical clustering, feature ratio values were calculated by averaging the median within-array spot replicate measures between replicate arrays. Where appropriate, replicate variation was used to weight feature measurements. Hierarchical clustering was performed using the C Clustering Library version 1.32 [36]. Data were clustered using average linkage and Pearson correlation as the distance measure.

All microarray data are available at the Gene Expression Omnibus (GEO) repository at the National Center for Biotechnology Information (http://www.ncbi.nlm.nih.gov/geo).

## Supporting Information

Figure S1 Analysis of Splice Site Sequences and Transcript Behavior in Comparisons between *prp2-1, prp8-1,* and *prp5-1*
Purple hashes denote the locations of introns with canonical and noncanonical 5′ and 3′ splice sites and branch point sequences.
(5.0 MB EPS)

Figure S2 Validation of Microarrays Using Quantitative RT-PCR (A) Quantitative RT-PCR (QPCR) experiments comparing wild-type samples with *prp2-1* (blue), *prp8-1* (red), or *prp5-1* (green) samples. Input material was normalized using the average value obtained from probes targeting both NRG1 and ECO1 transcripts. Values shown are derived from triplicate measurements with error bars included.
(B) Comparison of QPCR and microarray values for genes shown in (A).
(1.4 MB EPS)

Figure S3 The Extent of Precursor Accumulation Is Independent of Initial Precursor Levels Ratio values corresponding to the precursor species of the indicated genes (top) derived from each of three mutant analyses are plotted against initial precursor values as determined in Table 1.
(1.3 MB EPS)

Figure S4 Splicing Mutants Examined Flow diagram of splicing and spliceosomal recycling pathways showing putative stage of action of each interrogated allele. Alleles in factors involved in 3′ end processing and mRNA nuclear export were also tested in this study.
(1.2 MB EPS)

Figure S5 Individual Splicing Profiles of Each Mutant Examined (2.6 MB EPS)

Figure S6 High-Throughput Sample Collection and Preparation (A) Experimental time courses of temperature shifts were performed in a 96-well format. Samples were from transferred from flasks at 25 °C to a 96-well plate shaking in a 37 °C bath in a reverse time course. Matched wild-type and mutant samples were collected in the same 96-well plate to ensure consistency in time points between hybridized samples (right half and left half of the plate, respectively). Samples were collected for two biological dye-flipped array replicates in each 96-well plate (top half and bottom half of the plate).
(B) Replicate samples of total RNA from wild-type cells grown to optical densities between A_600_ = 0.5 and A_600_ = 0.7 at 30 °C purified using either the standard or the high-throughput method (see Materials and Methods) are shown here run on a 1% agarose gel stained with ethidium bromide.
(C) RT efficiency of total RNA samples shown in (B).
(4.2 MB EPS)

Figure S7 Design Scheme for Mature mRNA Probes To design mature mRNA probes (see Figure 2, probe M), a range of possible target-binding energies between probe sequence and exon sequence flanking exon–exon junctions were experimentally tested to determine what binding energy would yield the highest signal to noise. Sample probe sequences for three of the 12 tested targets are shown here.
(1.2 MB PDF)

Figure S8 Microarray Design Each biological sample is analyzed with three layers of data replication. Each probe is printed on the arrays three times by each of two different printing pins. Within a block (printed by a single printing pin) the three spot replicates are printed in the same column, separated vertically by eight rows. This vertical separation allows for assessment of top-to-bottom spatial spot ratio and intensity bias within each block. Block replicates (printed by two different pins) are oriented on the array with 180° rotational symmetry (for example, block 5 and 12). Horizontal and vertical separation of blocks allows for between-block assessment of both top-to-bottom and left-to-right spatial spot ratio and intensity bias. Finally, each experiment is assayed on two dye-flipped biological replicate arrays.
(7.0 MB PDF)

Figure S9 Global Quality Assessment for an Individual Microarray An example of a standard qualitative assessment of array quality, with array data from the *prp8-1* versus wild-type 30-min 37 °C temperature shift.
(A) Prenormalization spot log_2_ ratios (M-values) compared to total spot intensities (A-values), with traces showing the average behavior of each of the 16 blocks corresponding to individual printing pins.
(B) Spatial visualization of prenormalization M-value ranks across the array.
(C) Hexagon binning plot showing distribution and density of global Loess normalized M-values versus A-values.
(D) Spatial visualization of global Loess normalized M-values across the array.
(2.3 MB EPS)

Figure S10 Quality Assessment of Within-Array and Between-Array Replicate Agreement (A) Dotplot of normalized, unfiltered, log_2_ ratios (M-values) for every probe on the *prp8-1* versus wild-type 30-min 37 °C temperature shift array. Each set of six replicate probes is plotted on a single line vertically. M-values for probes printed in the first set of printing blocks (blocks 1–8) are plotted in blue, while M-values for probes printed in the second set of printing blocks (blocks 9–16) are plotted in orange. Spot replicate sets are arranged vertically based on the median M-value of each set.
(B) Density trace (Gaussian smoothing) of standard deviations for each replicate set on the array shown in (A). Three different density traces are shown for the three different types of probe sets.
(C) Plot of normalized M-values on the *prp8-1* versus wild-type 30-min 37 °C temperature shift array (Exp/Ref) and the matched dye-flipped replicate array (Ref/Exp). Points corresponding to total mRNA features, pre-mRNA features, and mature mRNA features are plotted in blue, red, and green, respectively. Legend shows Pearson correlations between the values for each of these types of features in the two arrays.
(2.6 MB EPS)

Table S1 Oligonucleotide Sequences Used in QPCR Experiments (14 KB XLS)

Table S2 Evidence for Differential Expression in the *prp2-1* Mutant Evidence for differential expression of intron sequences after shifting cells carrying the temperature sensitive *prp2-1* mutation versus wild-type cells to 37 °C for 30 min. The “ORF Name” column lists the standard yeast ORF name associated with each intron. M values represent averaged, log_2_-transformed ratios. The B-statistic represents the log posterior odds ratio.
(46 KB XLS)

Table S3 Evidence for Differential Expression in the *prp8-1* Mutant Evidence for differential expression of intron sequences after shifting cells carrying the temperature sensitive *prp8-1* mutation versus wild-type cells to 37 °C for 30 min. The “ORF Name” column lists the standard yeast ORF name associated with each intron. M values represent averaged, log_2_-transformed ratios. The B-statistic represents the log posterior odds ratio.
(46 KB XLS)

Table S4 Evidence for Differential Expression in the *prp5-1* Mutant Evidence for differential expression of intron sequences after shifting cells carrying the temperature sensitive *prp5-1* mutation versus wild-type cells to 37 °C for 30 minutes. The “ORF Name” column lists the standard yeast ORF name associated with each intron. M values represent averaged, log_2_-transformed ratios. The B-statistic represents the log posterior odds ratio.
(46 KB XLS)

Table S5 Evidence for Differential Expression in the *prp8-101* Mutant Evidence for differential expression of intron sequences after shifting cells carrying the temperature sensitive *prp8-101* mutation versus wild-type cells to 37° C for 30 min. The “ORF Name” column lists the standard yeast ORF name associated with each intron. M values represent averaged, log_2_-transformed ratios. The B-statistic represents the log posterior odds ratio.
(46 KB XLS)

## Abbreviations

pre-mRNA: pre-messenger RNA
RPG: ribosomal protein gene
snRNA: small nuclear RNA

## Note Added in Proof

Reference [38] is cited out of order because it was added while the article was in proof.
