# Supplementary material for: Transcript Specificity in Yeast Pre-mRNA Splicing Revealed by Mutations in Core Spliceosomal Components
Source: PLoS Biol. 2007 Mar 27;5(4):e90. doi: 10.1371/journal.pbio.0050090 (PMC1831718; doi:10.1371/journal.pbio.0050090)
Supplement: Supplementary file 1 [file pbio.0050090.sg007.pdf]

Figure S7

YML05C

Exon 1 3' end	energy	length	energy	Exon 2 5' end
GGTTTCTCTGGCTTCCCAGTTACTG	33	25	32.1	AAGACGGTAAGTGTCCAGGTAA
GTTTCTCTGGCTTCCCAGTTACTG	30.9	24	30.9	AAGACGGTAAGTGTCCAGGTAA
TTTCTCTGGCTTCCCAGTTACTG	29.4	23	29.4	AAGACGGTAAGTGTCCAGGTAA
TTCTCTGGCTTCCCAGTTACTG	28.2	22	27.9	AAGACGGTAAGTGTCCAGGTAA
TCTCTGGCTTCCCAGTTACTG	27	21	26.7	AAGACGGTAAGTGTCCAGGTA
CTCTGGCTTCCCAGTTACTG	25.5	20	25.8	AAGACGGTAAGTGTCCAGGT
TCTGGCTTCCCAGTTACTG	24	19	24.3	AAGACGGTAAGTGTCCAGG
CTGGCTTCCCAGTTACTG	22.5	18	22.2	AAGACGGTAAGTGTCCAG
TGGCTTCCCAGTTACTG	21	17	20.7	AAGACGGTAAGTGTCCA
GGCTTCCCAGTTACTG	19.3	16	19	AAGACGGTAAGTGTCC
GCTTCCCAGTTACTG	17.2	15	16.9	AAGACGGTAAGTGTC
CTTCCCAGTTACTG	14.9	14	15.4	AAGACGGTAAGTGT
TTCCCAGTTACTG	13.4	13	13.9	AAGACGGTAAGTG
TCCCAGTTACTG	12.2	12	12.2	AAGACGGTAAGT
CCCAGTTACTG	10.7	11	10.7	AAGACGGTAAG
CCAGTTACTG	8.6	10	9.2	AAGACGGTAA
CAGTTACTG	6.5	9	8	AAGACGGTA
AGTTACTG	4.8	8	7.1	AAGACGGT

snR17A

Exon 1 3' end	energy	length	energy	Exon 2 5' end
TTAAAATCTGTGTCGACGTACTTCA	30.9	25	28.6	TAGGATCATTTCTATAGGAATCG
TAAAATCTGTGTCGACGTACTTCA	29.7	24	27.1	TAGGATCATTTCTATAGGAATCG
AAAATCTGTGTCGACGTACTTCA	28.8	23	25.6	TAGGATCATTTCTATAGGAATCG
AAATCTGTGTCGACGTACTTCA	27.6	22	22.8	TAGGATCATTTCTATAGGAATC
AATCTGTGTCGACGTACTTCA	26.4	21	21.3	TAGGATCATTTCTATAGGAAT
ATCTGTGTCGACGTACTTCA	25.2	20	20.4	TAGGATCATTTCTATAGGAA
TCTGTGTCGACGTACTTCA	24.3	19	19.2	TAGGATCATTTCTATAGGA
CTGTGTCGACGTACTTCA	22.8	18	17.7	TAGGATCATTTCTATAGG
TGTGTCGACGTACTTCA	21.3	17	15.6	TAGGATCATTTCTATAG
GTGTCGACGTACTTCA	19.6	16	14.1	TAGGATCATTTCTATA
TGTCGACGTACTTCA	18.1	15	13.2	TAGGATCATTTCTAT
GTCGACGTACTTCA	16.4	14	12.3	TAGGATCATTTCTA
TCGACGTACTTCA	14.9	13	11.4	TAGGATCATTTCT
CGACGTACTTCA	13.4	12	9.9	TAGGATCATTTC
GACGTACTTCA	10.6	11	8.4	TAGGATCATTT
ACGTACTTCA	9.1	10	7.2	TAGGATCATT
CGTACTTCA	7.6	9	6	TAGGATCAT
GTACTTCA	4.8	8	5.1	TAGGATCA

YKL081C

Exon 1 3' end	energy	length	energy	Exon 2 5' end
GAAGCTTTGGCTATCCAATTTTATT	28.8	25	32.3	TGGCTAATCAAGTTGCCGATGAAAA
AAGCTTTGGCTATCCAATTTTATT	27.3	24	31.1	TGGCTAATCAAGTTGCCGATGAAA
AGCTTTGGCTATCCAATTTTATT	26.1	23	29.9	TGGCTAATCAAGTTGCCGATGAA
GCTTTGGCTATCCAATTTTATT	24.6	22	28.7	TGGCTAATCAAGTTGCCGATGA
CTTTGGCTATCCAATTTTATT	22.3	21	27.2	TGGCTAATCAAGTTGCCGATG
TTTGGCTATCCAATTTTATT	20.8	20	25.5	TGGCTAATCAAGTTGCCGAT
TTGGCTATCCAATTTTATT	19.6	19	24.6	TGGCTAATCAAGTTGCCGA
TGGCTATCCAATTTTATT	18.4	18	23.1	TGGCTAATCAAGTTGCCG
GGCTATCCAATTTTATT	16.7	17	20.3	TGGCTAATCAAGTTGCC
GCTATCCAATTTTATT	14.6	16	18.2	TGGCTAATCAAGTTGC
CTATCCAATTTTATT	12.3	15	15.9	TGGCTAATCAAGTTG
TATCCAATTTTATT	10.8	14	14.2	TGGCTAATCAAGTT
ATCCAATTTTATT	9.9	13	13	TGGCTAATCAAGT
TCCAATTTTATT	9	12	11.5	TGGCTAATCAAG
CCAATTTTATT	7.5	11	10	TGGCTAATCAA
CAATTTTATT	5.4	10	8.8	TGGCTAATCA
AATTTTATT	3.7	9	7.1	TGGCTAATC
ATTTTATT	2.5	8	5.6	TGGCTAAT
