# Supplementary material for: Transcript Specificity in Yeast Pre-mRNA Splicing Revealed by Mutations in Core Spliceosomal Components
Source: PLoS Biol. 2007 Mar 27;5(4):e90. doi: 10.1371/journal.pbio.0050090 (PMC1831718; doi:10.1371/journal.pbio.0050090)
Supplement: Supplementary file 2 [file pbio.0050090.sg008.pdf]

Figure S8

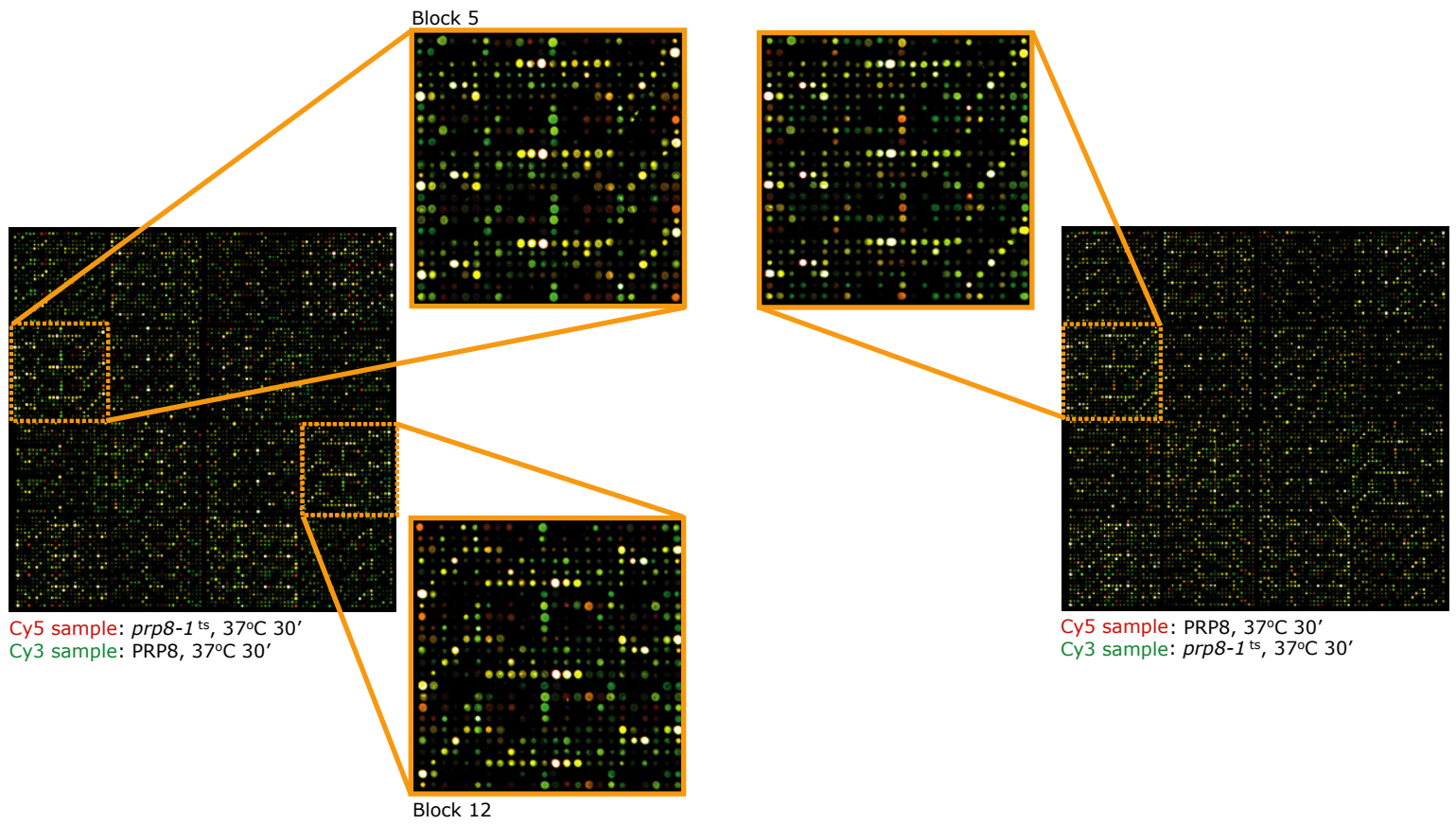

Block 5
Block 12
Cy5 sample: *prp8-1*[ts], 37ºC 30′
Cy3 sample: PRP8, 37ºC 30′
Cy5 sample: PRP8, 37ºC 30′
Cy3 sample: *prp8-1*[ts], 37ºC 30′
