# Supplementary material for: Transcript Specificity in Yeast Pre-mRNA Splicing Revealed by Mutations in Core Spliceosomal Components
Source: PLoS Biol. 2007 Mar 27;5(4):e90. doi: 10.1371/journal.pbio.0050090 (PMC1831718; doi:10.1371/journal.pbio.0050090)
Supplement: Supplementary file 3 [file pbio.0050090.sg010.pdf]

# Figure S10

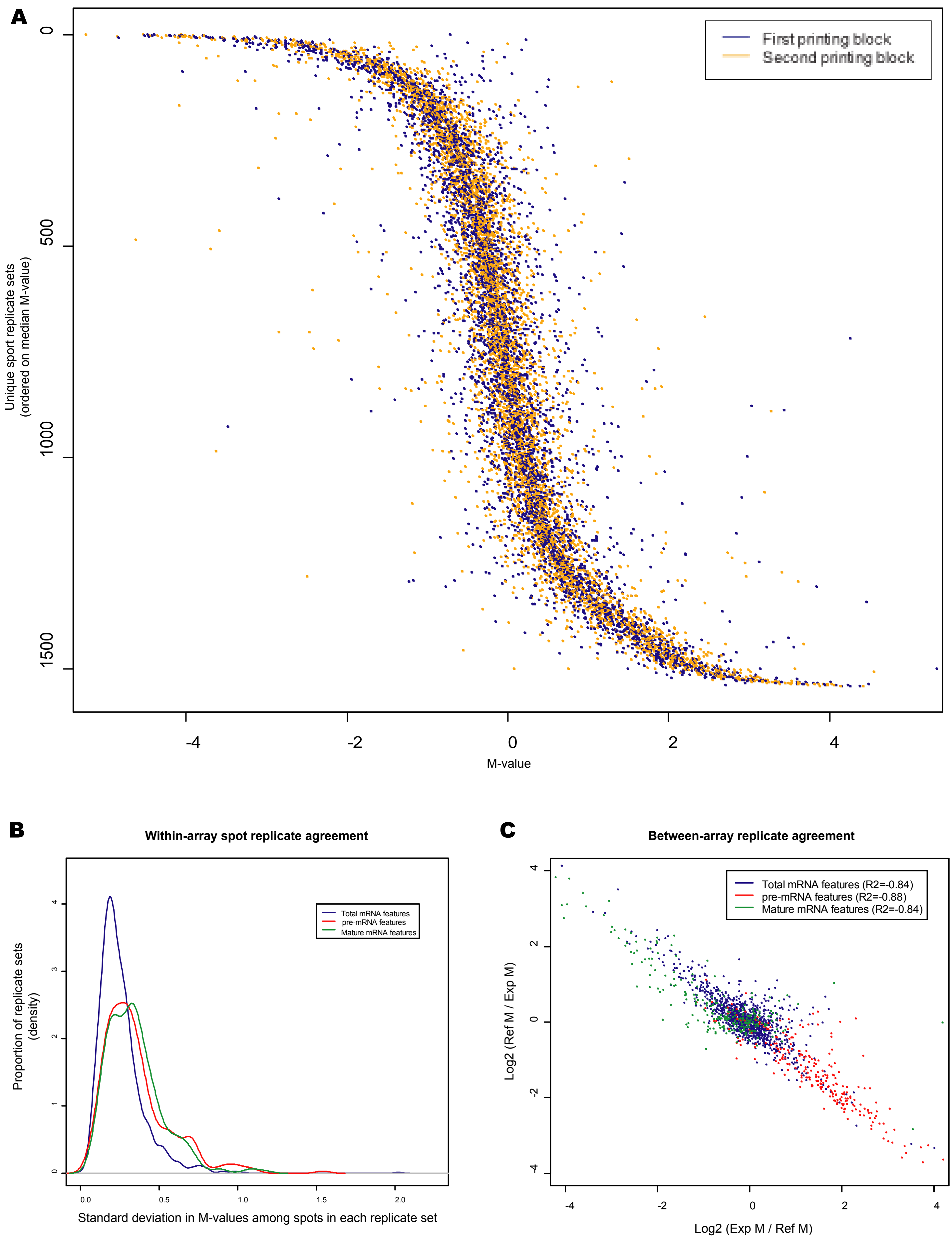
A
First printing block
Second printing block
Unique sport replicate sets
(ordered on median M-value)
0
500
1000
1500
-4
-2
0
2
4
M-value
B
Within-array spot replicate agreement
Total mRNA features
pre-mRNA features
Mature mRNA features
Proportion of replicate sets
(density)
Standard deviation in M-values among spots in each replicate set
0.0
0.5
1.0
1.5
2.0
C
Between-array replicate agreement
Total mRNA features (R2=-0.84)
pre-mRNA features (R2=-0.88)
Mature mRNA features (R2=-0.84)
Log2 (Ref M / Exp M)
Log2 (Exp M / Ref M)
